# Supplementary material for: A Global Screen for Assembly State Changes of the Mitotic Proteome by SEC-SWATH-MS
Source: Cell Syst. 2020 Feb 26;10(2):133–155.e6. doi: 10.1016/j.cels.2020.01.001 (PMC7042714; doi:10.1016/j.cels.2020.01.001)

Q14689 | DIP2A_HUMAN | DIP2A C21orf106 DIP2 KIAA0184
Monomer MW [kDa]: 170.369 Monomer expected elution fraction: 36

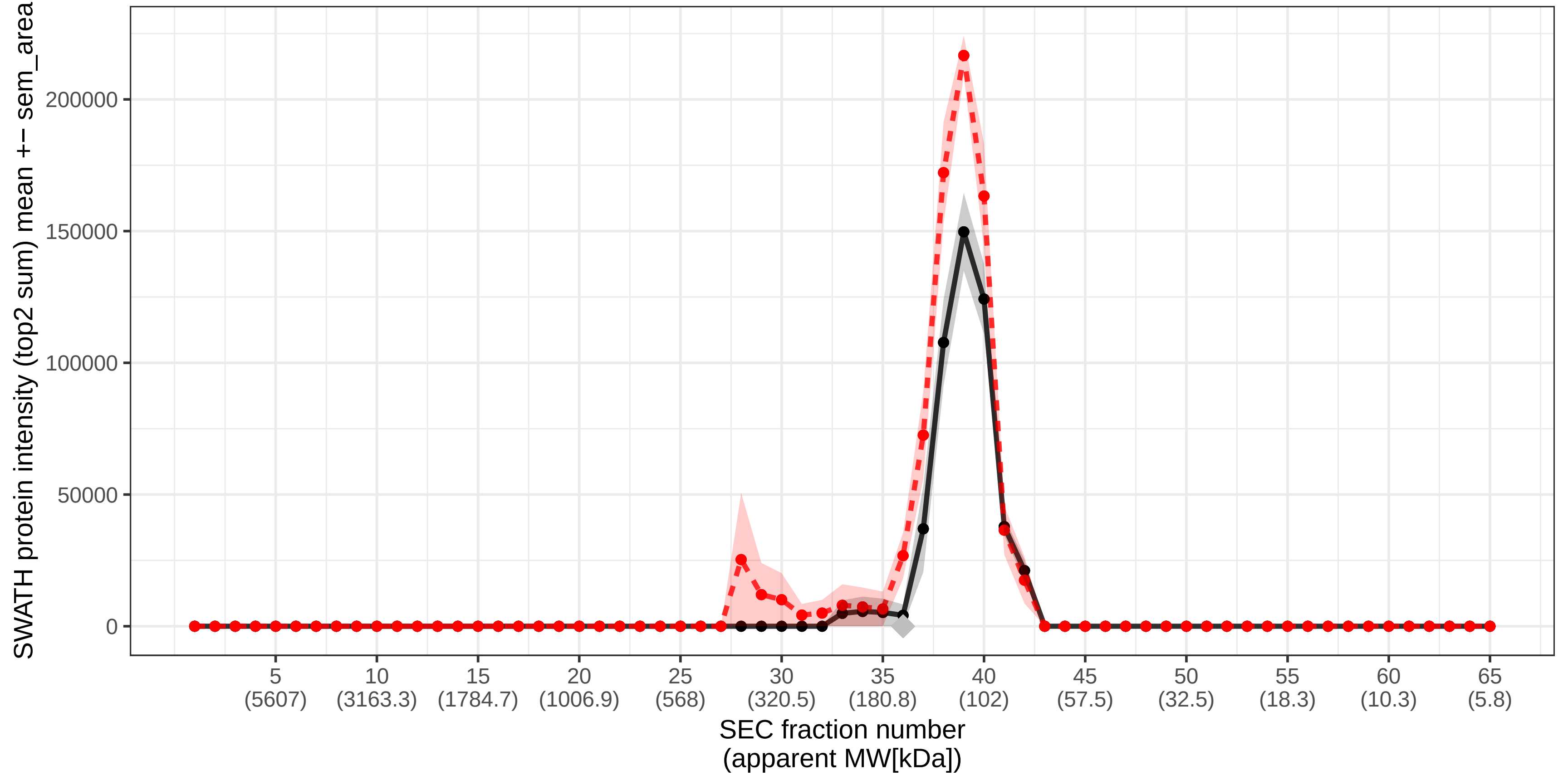

Supplement: Data S1. SEC-SWATH-MS Protein Chromatograms, Related to Figure 1 [file mmc6.zip › SECchrom_Q14689_DIP2A_HUMAN_DIP2A_C21orf106_DIP2_KIAA0184.pdf]
